# Encaminhamento de Angina Refratária para Reabilitação Cardiovascular: Um Paciente Negligenciado

**DOI:** 10.36660/abc.20220695

**Published:** 2022-11-01

**Authors:** Mauricio Milani, Juliana Goulart Prata Oliveira Milani, Gerson Cipriano

**Affiliations:** 1 Universidade de Brasília Brasília DF Brasil Programa de Pós-Graduação em Ciências e Tecnologias da Saúde, Universidade de Brasília (UnB), Brasília, DF – Brasil; 2 Clínica Fitcordis Medicina do Exercício Brasília DF Brasil Clínica Fitcordis Medicina do Exercício, Brasília, DF – Brasil; 3 Universidade de Brasília Brasília DF Brasil Programa de Ciências da Reabilitação, Universidade de Brasília (UnB), Brasília, DF – Brasil

**Keywords:** Doença Arterial Coronariana, Reabilitação Cardíaca, Exercício, Isquemia Miocárdica/tratamento farmacológico, Angina Estável

A reabilitação cardiovascular (RC) é um tratamento eficaz e seguro para pacientes com doença arterial coronariana (DAC) estável,^1^ com benefícios estabelecidos para melhorar a qualidade de vida e reduzir a mortalidade cardiovascular e a internação hospitalar.^2^ Os efeitos da RC na redução da isquemia miocárdica têm sido documentados,^3–5^ justificando a sua recomendação Classe IA.^1^ No entanto, a RC ainda é negligenciada e subutilizada em todo o mundo.^6^

A angina refratária (AR) é uma condição incapacitante que afeta pacientes com DAC sob terapia medicamentosa otimizada, com carga isquêmica residual por mais de três meses e inelegíveis para intervenções de revascularização. A AR está associada à redução da qualidade de vida, limitação ao exercício e distúrbios biopsicossociais. Idealmente, o manejo clínico deve ser orientado por centros especializados com o objetivo de otimizar múltiplas terapias farmacológicas e avaliar opções de intervenção.^7^ Nesse contexto, a RC abrangente é um tratamento valioso para a AR, considerando sua abordagem multidisciplinar, incluindo gerenciamento de fatores de risco, apoio psicológico e treinamento físico,^8^ embora estes últimos possuam evidências limitadas.^1^

Há uma década, Asbury et al.,^9^ demonstraram em um estudo piloto controlado randomizado os benefícios de uma RC na melhora da capacidade física, sem comprometer dor comórbida, angina ou risco de um evento adverso grave em 42 pacientes com AR. No entanto, neste estudo, a capacidade física foi avaliada pelo teste Shuttle Walk, e a prescrição da intensidade do exercício (60-75% da reserva de frequência cardíaca prevista para a idade ou 40-60% se insuficiência cardíaca) foi diferente da recomendação das diretrizes atuais,^1,10^ ressaltando a necessidade de estudos na área.

Apesar dos potenciais benefícios, os pacientes com AR geralmente não são encaminhados para RC devido à apreensão de eventos adversos durante o exercício físico,^1^ principalmente relacionados ao desencadeamento de isquemia miocárdica durante o treinamento físico. Por outro lado, Noel et al.,^11^ demonstraram que o treinamento isquêmico prolongado e repetido pode ser bem tolerado sem evidência de lesão miocárdica, arritmias significativas ou disfunção ventricular esquerda. No entanto, este estudo não se concentrou na AR, mas em 22 pacientes com DAC. Da mesma forma, a real prevalência de desencadeamento de isquemia miocárdica durante uma sessão de RC pode estar subestimada, pois alguns estudos já demonstraram uma prevalência de 54 a 81% de isquemia cintilográfica silenciosa durante o treinamento físico em pacientes com DAC com carga de isquemia residual, embora sem eventos adversos secundários.^5,12,13^

Nesse contexto, é importante destacar os achados do estudo intitulado “A lesão miocárdica ocorre após uma sessão aguda de exercício aeróbico em pacientes com angina refratária?”^14^ Este estudo teve como objetivo avaliar o efeito de uma sessão aguda de exercício aeróbico nos níveis de Troponina T cardíaca de alta sensibilidade (hs-cTnT) em 32 pacientes com AR com classe funcional (CCS) acima de II e isquemia miocárdica documentada por ecocardiograma de estresse. Neste estudo, a intensidade do exercício foi determinada de acordo com um teste de exercício cardiopulmonar prévio (TECP), um método padrão-ouro para prescrição de exercício.^10^ A sessão de exercício foi realizada em esteira rolante com intensidade de exercício monitorada pela frequência cardíaca de acordo com o primeiro limiar ventilatório ou limiar de angina. Uma dor de angina de até 3 em uma escala de 0-10 (leve a moderada) foi permitida durante o treinamento de exercício agudo, e os níveis de hs-cTnT foram determinados 3 horas após a sessão.^14^

O principal achado do estudo foi que as dosagens de hs-cTnT não revelaram diferenças significativas antes e após uma sessão de exercício, embora 53,1% dos pacientes tenham apresentado sintomas de angina durante o exercício, mas sem alterações eletrocardiográficas. Além disso, não houve eventos adversos ao longo do estudo, e os autores concluíram que o protocolo de exercícios era seguro para pacientes com AR.^14^

Embora estimulante e extremamente promissora, a conclusão foi baseada em uma única sessão de exercício de um estudo clínico não randomizado e não controlado,^14^ destacando mais uma vez a demanda por mais pesquisas sobre o tema. Os autores podem ter dados adicionais a serem publicados no futuro, pois o estudo está registrado no *Clinical Trials* (NCT03218891) como intervencionista. Estamos ansiosos por esses próximos resultados para permitir o aumento da recomendação e o nível de evidência de RC em pacientes com AR em futuras diretrizes.^1^

Até então, a segurança da RC demonstrada por este estudo^14^ e a segurança e eficácia preliminares demonstradas por Asbury et al.,^9^ devem ser reforçados, estimulando a indicação de RC para pacientes com angina refratária, visando os potenciais benefícios já demonstrados no amplo espectro da DAC, revertendo o encaminhamento anteriormente negligenciado.
