# Supplementary material for: Encaminhamento de Angina Refratária para Reabilitação Cardiovascular: Um Paciente Negligenciado
Source: Arq Bras Cardiol. 2022 Nov 1;119(5):754–5. [Article in Portuguese] doi: 10.36660/abc.20220695 (PMC9750214; doi:10.36660/abc.20220695)
Supplement: Translation

# Translation: Refractory Angina Referral to Cardiovascular Rehabilitation: A Neglected Patient

**Authors:** Mauricio Milani, Juliana Goulart Prata Oliveira Milani, Gerson Cipriano

**Affiliations:** 1 Brasília DF Brazil Programa de Pós-Graduação em Ciências e Tecnologias da Saúde, Universidade de Brasília (UnB), Brasília, DF – Brazil; 2 Brasília DF Brazil Clínica Fitcordis de Medicina do Exercício, Brasília, DF – Brazil; 3 Brasília DF Brazil Programa de Ciências da Reabilitação, Universidade de Brasília (UnB), Brasília, DF – Brazil

**Keywords:** Coronary Artery Disease, Cardiac Rehabilitation, Exercise, Myocardial Ischemia/drug therapy, Angina, Stable

Cardiovascular rehabilitation (CR) is an effective and safe treatment for stable coronary artery disease (CAD) patients,^1^ with established benefits to improving quality of life and reducing cardiovascular mortality and hospital admission.^2^ The effects of CR on reducing myocardial ischemia have been documented,^3–5^ justifying the CR Class IA recommendation.^1^ However, CR is still neglected and underused worldwide.^6^

Refractory angina (RA) is a disabling condition affecting CAD patients under optimized medical therapy with a residual ischemic burden for more than three months who are ineligible for revascularization interventions. RA is associated with reduced quality of life, exercise limitation, and biopsychosocial disorders. Ideally, clinical management should be guided by specialized centers aiming to optimize multiple pharmacologic therapies and evaluated interventional options.^7^ In this context, comprehensive CR is a valuable treatment for RA considering its multi-component approach, including risk factor management, psychological support, and exercise training,^8^ although the latter possess limited evidence.^1^

A decade ago, Asbury et al.^9^ demonstrated in a randomized controlled pilot study the benefits of a CR in improving physical ability without compromising comorbid pain, angina, or risk of a severe adverse event in 42 RA patients. However, in this study, the physical capacity was assessed by the Shuttle Walk test, and the exercise intensity prescription (60-75% of age-predicted heart rate reserve or 40-60% if heart failure) was different from the current guidelines recommendation,^1,10^ stressing the necessity for studies in the area.

Despite the potential benefits, patients with RA are generally not referred to CR due to apprehension of adverse events during physical exercises,^1^ mainly related to myocardial ischemia triggering during exercise training. Conversely, Noel et al.^11^ demonstrated that prolonged and repeated ischemic training could be well tolerated without evidence of myocardial injury, significant arrhythmias, or left ventricular dysfunction. However, this study focused not on RA but 22 CAD patients. Likewise, the actual prevalence of myocardial ischemia triggering during a CR session might be underestimated since some studies have already demonstrated a 54 to 81% prevalence of silent scintigraphic ischemia during exercise training in CAD patients with residual ischemia burden, although with no secondary adverse events.^5,12,13^

In this context, it is important to highlight the findings from the study entitled “Does myocardial injury occur after an acute aerobic exercise session in patients with refractory angina?”.^14^ This study aimed to assess the effect of an acute aerobic exercise session on high-sensitivity cardiac Troponin T (hs-cTnT) levels in 32 patients with RA with functional class (CCS) above II and myocardial ischemia documented by stress echocardiography. In this study, the exercise intensity was determined according to a previous cardiopulmonary exercise testing (CPET), a gold-standard method for exercise prescription.^10^ The exercise session was performed on a treadmill with exercise intensity monitored by heart rate according to the first ventilatory threshold or the angina threshold. An angina pain up to 3 on a 0-10 scale (mild to moderate) was allowed during the acute exercise training, and the levels of hs-cTnT were determined 3 hours after the session.^14^

The study's main finding was that hs-cTnT dosages did not reveal significant differences before and after an exercise session, although 53.1% of the patients had experienced angina symptoms during the exercise without electrocardiographic changes. Also, there were no adverse events throughout the study, and the authors concluded that exercise protocol was safe for patients with RA.^14^

Although exciting and extremely promising, the conclusion was based on a single exercise session of a non-randomized and non-controlled clinical study,^14^ once again highlighting the demand for further research on this topic. The authors may have additional data to be published in the future, as the study is registered in clinical trials (NCT03218891) as interventional. We are looking forward to these upcoming results to allow the increase of the recommendation and the level of evidence of CR in RA patients in future guidelines.^1^

Until then, the safety of CR demonstrated by this study^14^ and the preliminary safety and efficacy shown by Asbury et al.^9^ must be reinforced, stimulating the indication of CR to refractory angina patients by targeting the potential benefits already demonstrated in the broad spectrum of CAD, reversing the previously neglected referral.
